# Spontaneous spinal epidural hematoma: a case report

**DOI:** 10.1186/s12245-021-00379-0

**Published:** 2021-09-25

**Authors:** Ooi Chin Sheng, Ren-Chieh Wu, I-Hsin Chang

**Affiliations:** 1 Department of Medicine, Hualien Tzu Chi Hospital, Buddhist Tzu Chi Foundation, Tzu Chi University, Hualien, Taiwan; 2 Department of Emergency Medicine, Hualien Tzu Chi Hospital, Buddhist Tzu Chi Foundation, Hualien, Taiwan; 3 Program in Pharmacology and Toxicology, Tzu Chi University, 970 Hualien, Taiwan

**Keywords:** Lower limb weakness, Back pain, Spinal epidural hematoma

## Abstract

**Background:**

Spinal epidural hematomas usually occur under certain conditions; they rarely occur spontaneously. The prevalence of spontaneous spinal epidural hematoma is ~ 0.1 per 100,000, and the male-to-female ratio is approximately 1.4 to 1. Herein, we describe a rare case of spontaneous spinal epidural hematoma.

**Case presentation:**

A 63-year-old Taiwanese woman, with underlying hypertension, anemia, and a history of cardiovascular accident without sequela, was admitted to our emergency department with a chief complaint of sudden bilateral weakness in the lower limbs. Magnetic resonance imaging revealed a spontaneous epidural hematoma. The patient underwent emergency surgery to remove the epidural hematoma and laminectomy for decompression. The bilateral lower limb weakness was alleviated immediately after the surgery.

**Conclusion:**

In patients with no risk factors related to spinal epidural hematoma, symptoms of bilateral lower limb weakness must be investigated carefully because this condition may occur spontaneously.

## Background

Spinal epidural hematomas usually occur under certain conditions; they rarely occur spontaneously. The prevalence of spontaneous spinal epidural hematoma is ~0.1 per 100,000, and the male-to-female ratio is approximately 1.4 to 1 [1]. The case that we describe herein was one of these rare occurrences. Our purpose of presenting this case report is to warn physicians that lower limb weakness or back pain must be investigated for this condition, even though affected patients might not have any risk factors or any other possible cause.

## Case presentation

**Fig. 1:**
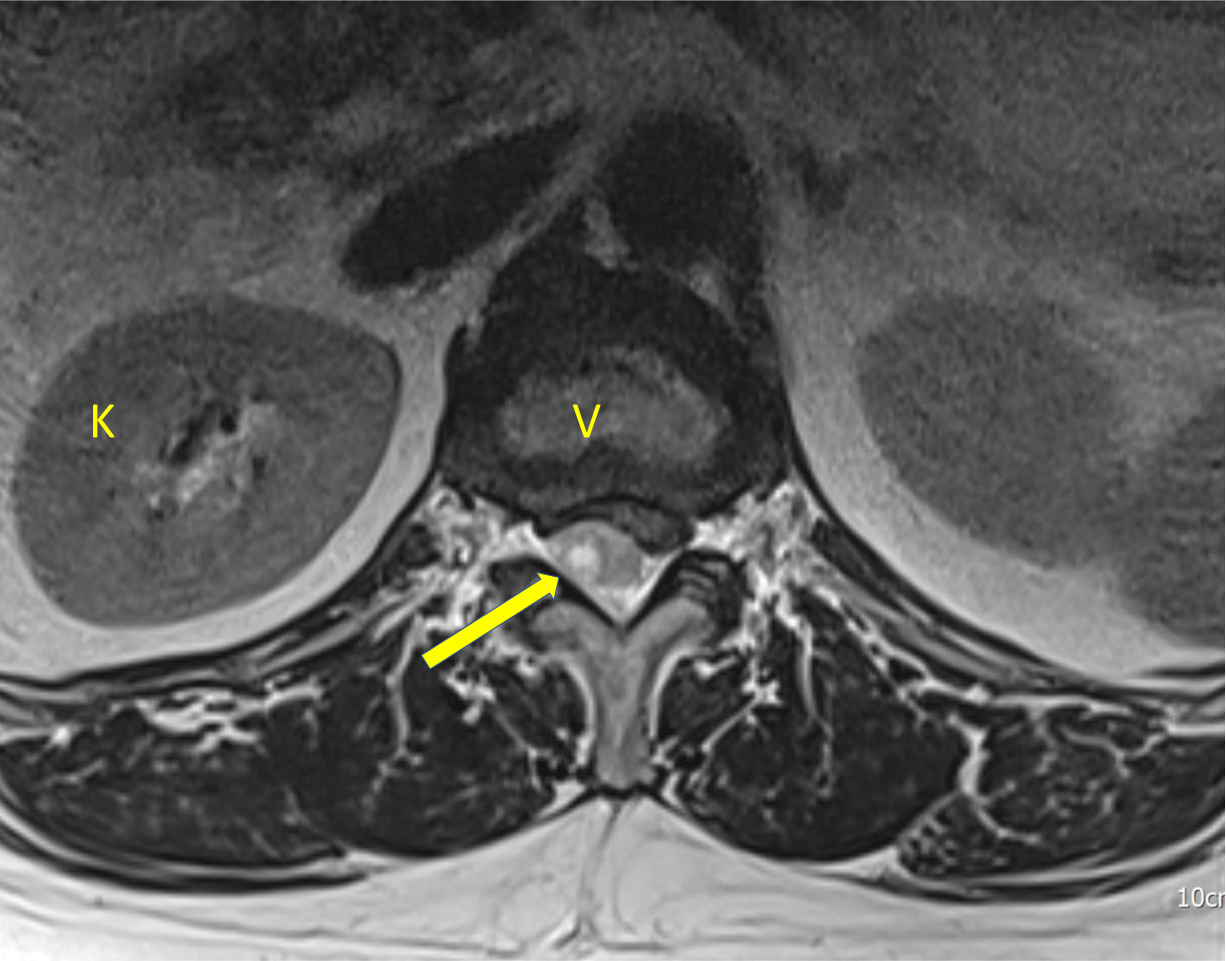
Lumbosacral magnetic resonance imaging without contrast, axial view. V: vertebrae, K: kidney, arrow: epidural hematoma occupies the spinal canal and compresses the spinal cord

**Fig. 2:**
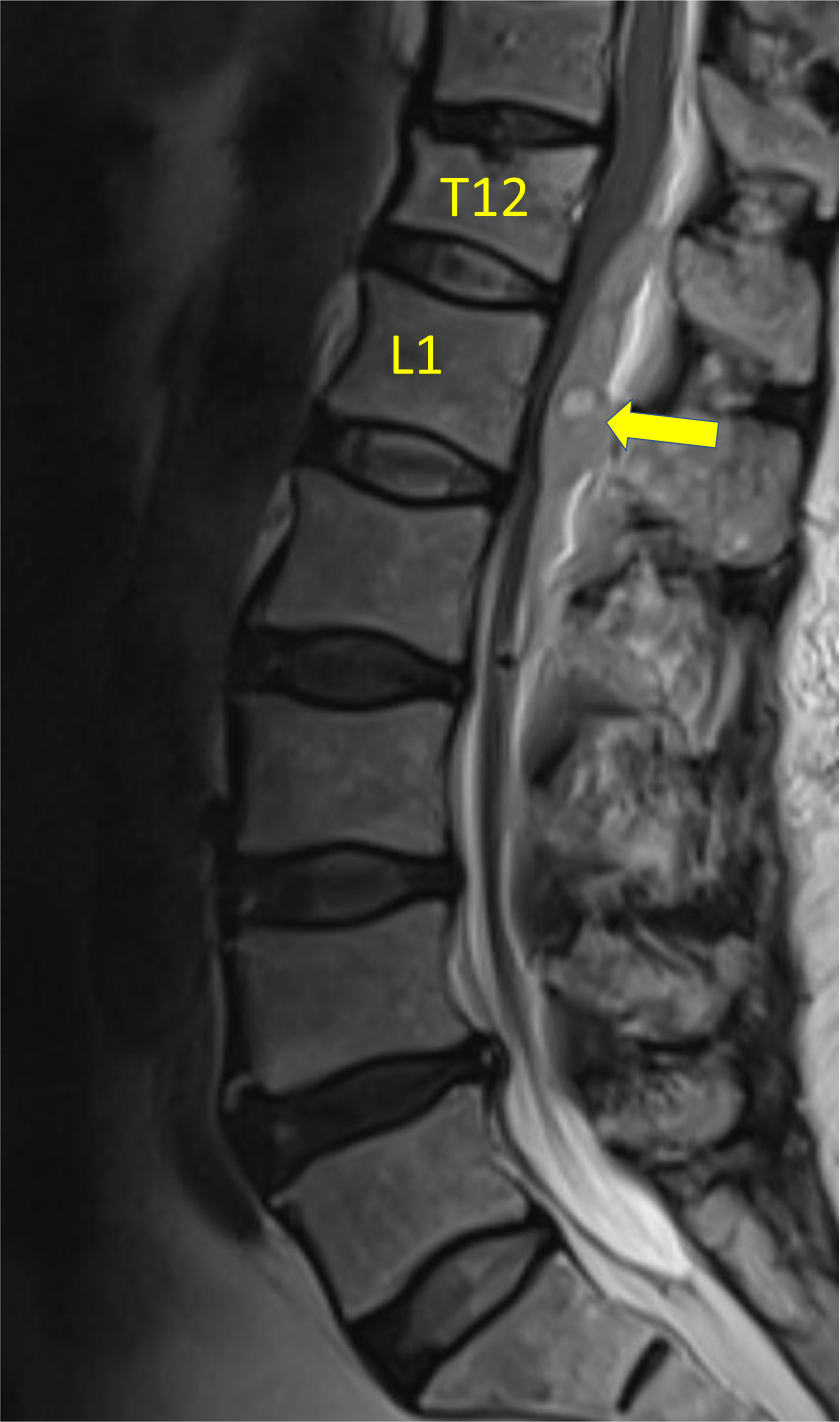
Lumbosacral magnetic resonance imaging without contrast, Sagittal view. T12: The 12th thoracic vertebrae, L1: The 1st lumbar vertebrae, arrow: an epidural hematoma extending from the levels of T12 to L2

A 63-year-old Taiwanese woman presented to our emergency department with sudden onset of weakness in both lower limbs. Her medical history included hypertension, anemia, and a cardiovascular accident without neurological sequelae. Two hours before admission, she experienced acute onset of bilateral lower back pain while bathing. She rated the pain as 5 out of 10 on the numeric rating scale (NRS). Approximately 30 min after her bath, while she was resting and watching television, she experienced sudden bilateral lower limb pain, weakness, and numbness. The numbness was described as the same feeling that follows electric shock. The back and lower limb pain worsened, and the NRS score rose from 5/10 to 10/10. She was unable to walk or even stand up because of the weakness and pain in both limbs. After calling the emergency number, she was sent to the regional hospital where magnetic resonance imaging of the thoracic spine to lumbar spine revealed an epidural hematoma extending from the levels of T12 to L1 (Figs. 1 and 2). Some analgesic medication was injected twice, but the pain persisted. She was then transferred to our medical center for further treatment.

During history taking, the patient denied any other discomfort such as nausea, vomiting, dizziness, headache, choking, seizure, focal neurological deficits (such as visual change or aphasia), urinary incontinence, fecal incontinence, fever, neck pain, abdominal pain, or any history of back trauma. Although this patient had a stroke ~ 12 years ago, she had recovered almost entirely after undergoing rehabilitation for 2 years, and she had consumed neither aspirin nor anticoagulant since then. She had undergone acupuncture 2 years earlier for her chronic sciatica. The patient did drink alcoholic beverages and chewed betel nuts over the previous 30 years, but she had never smoked.

During admission to our emergency department, her body temperature was 36.4 °C, respiratory rate was 18 per min, pulse rate was 67 bpm, blood pressure was 185/106 mm Hg, and oxygen saturation was 90% on room air. The physical examinations over the head, neck, chest, heart, and abdomen yielded normal findings. In both lower limbs, however, her muscle power was only approximately 3 (measured by Medical Research 58 Council Manual Muscle Testing scale). No edema or wound was present over the extremities. The deep tendon reflex was normal (2+) in both upper arms but decreased (1+) in both lower limbs. Tenderness over the back and both lower limbs was identified. Blood examinations did not show any significant findings and they were shown in Table 1.

**Table 1:**
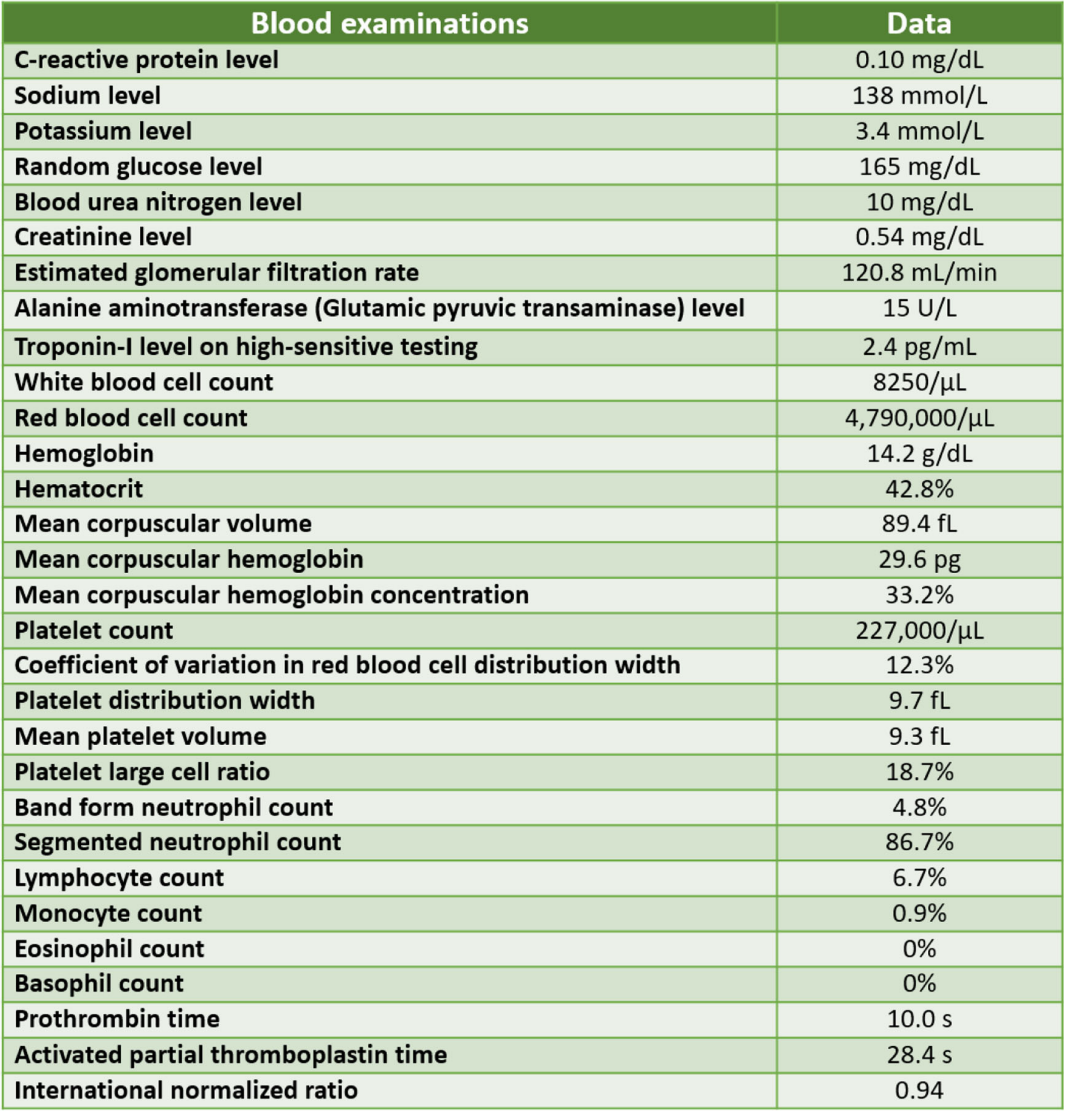
Blood examination results

For analgesic control, we initially prescribed celecoxib (Celebrex), 200 mg/cap BID; tramadol (Tramacet), 37.5 mg QHS; and acetaminophen, 325 mg QHS. However, her pain persisted; on the NRS, it remained as 9/10 or 10/10.

A neurosurgeon was consulted for the diagnosis of spinal epidural hematoma. The patient underwent emergency surgery to remove the epidural hematoma and laminectomy for decompression of T11–L3. After the operation, she was able to move her lower limbs with a greater range of motion, and the muscle power was ~ 4. The pain over the lower limbs was relieved, and the only pain she experienced was in the area of the surgical wound over the back. Acetaminophen for analgesic control was given for the wound pain. Tramadol was given occasionally for further pain control. Because the patient’s general condition was stable and she recovered well from the surgery without any neurological deficit, she was discharged from our hospital after 2 weeks. Physiotherapy for rehabilitation was recommended and the patient was well followed up indeed.

## Discussion and conclusions

Epidural hematoma can be classified as intracranial or spinal epidural hematoma. Epidural hematoma is defined as an “accumulation of blood in the potential space between dura and bone” [2]. Spinal epidural hematoma occurs most commonly after trauma, but, as our case shows, it may occur spontaneously. Its annual prevalence is ~ 1 per 1,000,000 [2].

Spinal epidural hematoma may develop after a lumbar puncture or epidural anesthesia. Other associated factors include thrombolysis, anticoagulation, thrombocytopenia, blood dyscrasias, neoplasms, coagulopathies, and vascular malformations [2]. Furthermore, some studies have shown that patients with a history of alcoholism or other forms of intoxication are at increased risk for epidural hematoma. In our case, the patient had been drinking alcohol beverages for ~ 30 years, as mentioned previously, but she denied any other history that might have been related to the common causes of spinal epidural hematoma.

The patient had undergone acupuncture ~ 2 years ago. Spinal acupuncture is a common and relatively safe analgesic treatment. Nevertheless, it may have produced serious side effects, such as direct spinal cord or nerve root injury, epidural abscesses, or subdural empyema. Moreover, some extremely rare cases of spinal epidural hematoma that occurred immediately after acupuncture have been reported [3]. No case of delayed spinal epidural hematoma occurring a few years after acupuncture has been reported. Because our patient had undergone acupuncture ~ 2 years ago, and because her general condition was stable after that, the acupuncture was probably not related to the spinal epidural hematoma.

The rate of mortality from epidural hematoma was reported to be 5–50% [2]. Therefore, treatment of spinal epidural hematoma is crucial. For patients with neurological deficits, immediate decompressive laminectomy and hematoma evacuation has been recommended [4]. The preoperative neurologic status may help predict the outcome of surgical treatment in a patient with spinal epidural hematoma [5]. Up to 42% of patients in whom this lesion caused incomplete sensorimotor deficit had complete sensorimotor recovery; the recovery rate was lower in those with incomplete sensory dysfunction, but was only 26% among patients with complete motor dysfunction and ~11% among those with complete sensorimotor dysfunction [6]. The patient exhibited incomplete sensorimotor dysfunction; hence, the prognosis after surgical treatment was good. As we expected, she was discharged without any neurological deficit 2 weeks after the surgery. We recommended further rehabilitation to ensure a full recovery.

In conclusion, symptoms of bilateral lower limb weakness must be investigated carefully, even if affected patients do not have any risk factors related to spinal epidural hematoma as this condition may occur spontaneously.

## Data Availability

Not applicable.

## Abbreviations

NRS: Numeric rating scale
